# Verteporfin suppresses the proliferation, epithelial-mesenchymal transition and stemness of head and neck squamous carcinoma cells via inhibiting YAP1

**DOI:** 10.7150/jca.34145

**Published:** 2019-07-10

**Authors:** Kui Liu, Shanmei Du, Peng Gao, Jie Zheng

**Affiliations:** 1 Department of Pathology, Medical School of Southeast University, Nanjing 210009, China; 2 Zibo Vocational Institute, Zibo 255314, China; 3 Division of Oncology and Center for Childhood Cancer Research, Children's Hospital of Philadelphia, Philadelphia, PA19104, USA; 4 Department of Biomedical and Health Informatics, Children's Hospital of Philadelphia, Philadelphia, PA 19104, USA

**Keywords:** Verteporfin, YAP1, Head & neck squamous cell carcinoma, Cell proliferation, Epithelial-mesenchymal transition, Stemness

## Abstract

**Purpose**: Yes-associated protein 1 (YAP1) is overexpressed in head and neck squamous cell carcinoma (HNSCC). However, it is unknown whether verteporfin, a YAP1 inhibitor, can inhibit HNSCC cells as well as the molecular mechanisms involved.

**Methods**: YAP1 expression was investigated by immunohistochemistry in human head and neck carcinoma tissues (n=70). CCK-8 assay, colony formation assay, flow cytometric analysis, wound-healing assay and Transwell migration and invasion assays were used to evaluated the effects of verteporfin on the six HNSCC cell lines (three HPV-positive and three HPV-negative). The transcription and protein expression levels of *YAP1* and its associated genes were investigated by real-time PCR and Western blotting, respectively. The effects of verteporfin on HNSCC cells *in vivo* were assessed by a xenograft model.

**Results**: YAP1 expression was significantly higher in carcinoma tissues than in tumor-adjacent normal tissues (n=10). A CCK-8 assay showed that the inhibitory effects of verteporfin on HNSCC cells were markedly enhanced by light activation. Verteporfin significantly inhibited HNSCC cell proliferation, migration and invasion, induced apoptosis, and arrested the cell cycle at the S/G2 phase. Verteporfin significantly attenuated the expression of genes related to epithelial-mesenchymal transition (*YAP1*, *Snail*, *CTNNB1* and *EGFR*) and stemness (*Oct4* and *YAP1*) and increased E-cadherin expression in HNSCC cells. Furthermore, verteporfin significantly inhibited PD-L1 expression in HNSCC cells. However, the expression levels of HPV-16 E6 and E7 did not change with VP treatment. The anticancer effect of verteporfin on HNSCC was confirmed by the inhibition of xenograft growth *in vivo*.

**Conclusions**: Our results indicate that YAP1 overexpression is involved in HNSCC tumorigenesis and verteporfin is a potential therapeutic drug for HNSCC.

## Introduction

Head and neck squamous cell carcinoma (HNSCC) is the 6th most common cancer worldwide 1. Recent studies have shown that more than 20% of HNSCCs contain human papillomavirus (HPV) DNA, especially that of HPV-16, in which E6 and E7 are major viral oncoproteins 2. HPV-positive and -negative HNSCCs represent different clinicopathological and molecular entities, and HPV-positive HNSCC patients have a better prognosis 3, 4. Because of the anatomical structures involved and the importance of organ function, HNSCC treatment is complicated, and the 5-year survival rate of patients has not improved significantly 5. Therefore, identifying new drugs and therapeutic approaches is required to improve the outcome of HNSCC therapy.

The Hippo pathway is an evolutionarily conserved signaling pathway regulating numerous biological processes, including cell growth, organ size, and tissue homeostasis. This pathway consists primarily of upstream signals, core kinases (MST1/2 and LATS1/2) and downstream effectors, including the transcriptional coactivators Yes-associated protein (YAP) and TAZ, which mainly interact with TEA domain (TEAD) family transcription factors (TFs) to regulate cell proliferation, survival, migration, stemness, epithelial-mesenchymal transition (EMT) and differentiation 6. The *YAP* gene encodes two major isoforms YAP1 and YAP2, which contain one WW domain and two WW domains, respectively.

Dysregulation of the Hippo pathway has been implicated in many human diseases, including cancer 6, 7. As a key component of the Hippo pathway, YAP has been found to be overexpressed in many human cancers, including HNSCCs 8-10. Therefore, YAP is an attractive therapeutic target in cancer. Verteporfin (VP), a YAP inhibitor, is FDA-approved for use with photodynamic therapy to treat age-related macular degeneration. VP has been recently proven to be an inhibitor of YAP-TEAD complex and preventing YAP-induced oncogenic growth 11. Recently, the anticancer activity of VP has been reported in various cancers, such as ovarian 11, colon 12, pancreatic 13 and thyroid 14 cancers. However, the effects of VP on HNSCC cells have seldom been reported and the anticancer mechanisms of VP are poorly understood. In this study, we aimed to investigate the effects of VP on cell proliferation, apoptosis, migration, invasion and the expression of certain key genes involved in the molecular biology of HNSCC and to assess the effects of VP on HNSCC cell xenografts.

## Materials and methods

### Human head and neck tissue array and immunohistochemical staining

The human head and neck carcinoma and normal tissue array, with stage and grade information, were purchased from Outdo Biotech Inc. (Shanghai, China). This array contained 70 carcinoma tissues and 10 tumor-adjacent normal tissues. The study was approved by the ethics committee of the Southeast University.

YAP1 protein expression in human head and neck tissues was detected by using peroxidase-based immunohistochemistry (IHC). In brief, formalin-fixed and paraffin-embedded tissue sections were deparaffinized in xylene and hydrated through descending concentrations of ethanol before being placed in blocking solution to inhibit endogenous peroxidase activity. The slides were incubated with primary antibody (1:200 dilution; Cell Signaling Technology, MA, USA) at 4°C overnight. A horseradish peroxidase-conjugated rabbit secondary antibody (1:4000 dilution; Proteintech, Rosemont, USA) was added for 60 min at room temperature, followed by 3,3′-diaminobenzidine kit (DAB, Invitrogen, Carlsbad, CA) for staining. Sections were scanned with an iSCAN Coreo slide scanner (3D-Histech, Pannoramic, Hungary). Positive YAP1 staining was defined as brown granules in the cytoplasm or nuclei. The intensity score was graded as follows: - (negative), + (low), ++ (moderate), and +++ (high). The results were evaluated by two independent pathologists.

### Cell lines and reagent

The sources and characteristics of the HPV-negative HNSCC cell lines SCC-4, CAL-27 and SCC-25 and the HPV 16-positive HNSCC cell lines UM-SCC-47, UPCI-SCC-090, and 93-VU-147T have been described in a previous publication 15. UM-SCC-47, UPCI-SCC-090 and 93-VU-147T cells were cultured in high glucose Dulbecco's Modified Eagle's Medium (H-DMEM) (HyClone). SCC-4, SCC-25 and CAL-27 cells were cultured in DMEM/F-12 (HyClone). All media were supplemented with 10% (v/v) fetal bovine serum (FBS) (Gibco-BRL), 100 units/ml penicillin and 100 μg/ml streptomycin (Beyotime Institute of Biotechnology, Shanghai, China).

VP (Selleck Chemicals, S1786) was dissolved in dimethyl sulfoxide (DMSO, Sigma-Aldrich) at a concentration of 10 mg/mL and stored at -80°C. During treatment, the stock solution was diluted to the required concentration using cell culture medium to yield the working solution in the dark.

### CCK-8 assay

The effects of VP on the proliferation of cancer cells were assessed using a CCK-8 kit (Beyotime) according to the manufacturer's manual, with or without light activation. Briefly, 2 × 10^3^ cells/well were seeded in 96-well plates, and allowed to attach overnight. Then the medium was replaced with fresh cell culture medium supplemented with various concentrations of VP and incubated in the dark. After 12 h, photoactivation was performed in the light-activated groups with a light (Philips three-base color straight fluorescent lamp, 14 watts) for 20 min, afterwords cells were cultured in a 37°C incubator. The groups without light activation were constantly maintained in the dark. Each group included six replicates.

The optical density (OD) at 450 nm was measured using a microplate reader (BioTek) at 24, 48 and 72 h. Cell viability was calculated as follows: Cell viability (%) = OD_treated group mean_/OD_control group mean_ × 100.

In the subsequent experiments, VP treatment was performed with light activation.

### Flow cytometric analysis

An apoptosis kit (MultiSciences Biotech, Hangzhou, China) and a cell cycle kit (Beyotime) were used according to the manufacturers' manuals. In the apoptosis assay, the cells were seeded in 6-well plates and VP treatment was as CCK-8 assay. After VP light activation for 24 h, the cells were harvested and washed twice with cold phosphate buffer saline (PBS) and stained with Annexin V-fluorescein isothiocyanate (FITC) and propidium iodide (PI) in the dark at room temperature for for 15 min. In the cell cycle assay, the cells were seeded in 6-well plates using non-serum cell culture medium for 12 h. Then the cells were treated with VP as CCK-8 assay. After VP light activation for 24 h, the cells were harvested, fixed in 70% ethanol at 4°C overnight, and then stained with PI at 4°C for 30 min in the dark. Stained cells were analyzed using a FACSCalibur flow cytometer (BD Biosciences).

### Colony formation assay

The cells (1 × 10^3^ cells/well) were plated in 6-well plates and allowed to attach overnight. Then the medium was replaced with fresh medium with VP for 12 h. Then, the cells were irradiated with the light for 20 min to activate the VP. The fresh medium was changed every three days. At 10 days, the cells were fixed with 4% paraformaldehyde for 30 min and stained with 0.1% crystal violet for 30 min. The cell colonies were imaged, and the number of colonies was counted for statistical analysis.

### Cell migration and invasion assays

For the wound-healing assay, cells were grown to confluence and treated with VP in the dark. After 12 h, the cells were irradiated with the light to activate VP. Next, the cell layer was scratched along the central axis using a sterile plastic tip, and the fresh medium was changed. The degree of cell migration at 24 h was calculated as follows: (Distance of cell migration at 24 h/width of scratch at 0 h) × 100%.

Transwell assay was used to assess the cell migration and invasive abilities. Briefly, after VP light activation, HNSCC cells (5 × 10^4^) were suspended in 0.1 mL of medium without FBS and seeded into the upper chambers of Matrigel-coated (BD Biosciences, Bedford, MA) (for assessing the cell invasion ability) or uncoated (for assessing the cell migration ability) polycarbonate membrane filters. Then, medium containing 10% FBS was added to the lower chambers as a chemoattractant. Cells that had migrated to the lower chambers at 24 h were fixed with 4% paraformaldehyde for 30 min and stained with 0.1% crystal violet for 30 min. Three low-magnification areas were randomly selected, and the number of migrated cells was counted.

### Reverse transcription and quantitative PCR (RT-qPCR)

The cells were seeded in 6-well plates using cell culture medium for 12 h. Then the cells were treated with VP in dark. After 12 h, the cells were irradiated with the light to activate VP. The cells were harvested after treatment with light activated VP for 12 h. Total RNA was extracted from cells using the RNAiso Plus (TAKARA Biotechnology, Dalian, China) according to the manufacturer's instructions. RT-qPCR was performed using a SYBR-Green-based PCR kit (TAKARA) with an Applied Biosystems StepOnePlus Real-Time PCR system (ABI, Foster City, CA, USA). The comparative Ct method was applied to determine the fold-differences in expression levels relative to those in β-actin. The primers used are listed in Table 1.

### Western blot analysis

The cells were prepared and treated with VP as RT-qPCR above. After VP light activation for 6 h or 12 h, total protein was extracted from cells for Western blot analysis as described previously 15. Antibodies against YAP1 (1:2000 dilution), E-cadherin (1:1000 dilution), Snail (1:1000 dilution), β-catenin (1:2000 dilution), EGFR (1:4000 dilution), programmed death ligand-1 (PD-L1) (1:1000 dilution), Twist1 (1:1000 dilution) and β-actin (1:4000 dilution) were obtained from Proteintech. An antibody against Oct4 (1:1000 dilution) was obtained from BioSS. Antibodies against HPV-16 E6 (1:1000 dilution) and E7 (1:200 dilution) were obtained from Abcam (Cambridge, UK) and Santa Cruz (CA, USA), respectively. A horseradish peroxidase-linked secondary antibody (1:4000 dilution) was obtained from Proteintech.

### Animal experiments

Six-week-old SCID mice (BALB/c) were handled according to the Guidelines for Animal Experiments of the Southeast University. Each of the nude mice was subcutaneously injected with 10^6^ cells/100 μl as indicated in Fig. 7. When the tumor volume reached approximately 80 mm^3^, the mice were randomized to the different experimental groups (n=4, half male and half female). VP was injected intraperitoneally at a concentration of 100 mg/kg or the vehicle (control) every 2 days. Tumors were excised and weighed at 21 days. Tumor volume was calculated as the product of all three dimensions.

### Statistical analysis

All experiments were repeated at least twice. The data were analyzed using GraphPad Prism version 5.0 (GraphPad Software, San Diego, CA, USA) and SPSS 17.0 (SPSS Inc., Chicago, IL) and expressed as the mean values ± SEM (standard error of the mean). Statistical analysis was performed using the standard Student's *t*-test. The significance of data obtained from patient specimens was determined using a Chi-square test. *P*<0.05 was considered to indicate a significant difference (*P< 0.05, **P≤ 0.01, ***P≤ 0.001).

## Results

### YAP1 expression was upregulated in HNSCC tissues

**Figure 1:**
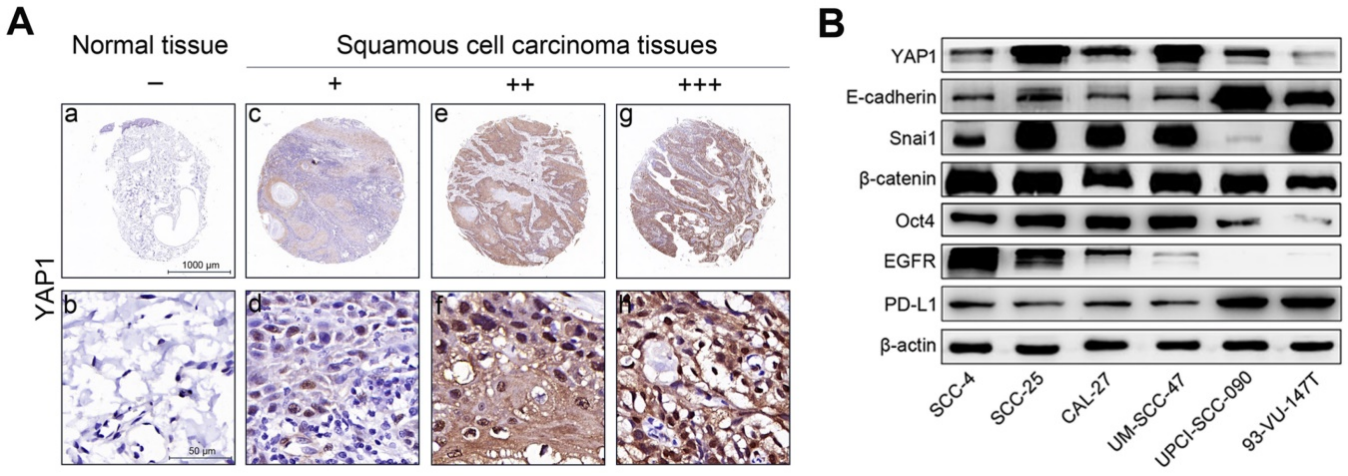
YAP1 expression in tissues and the expression levels of key proteins in HNSCC cells. (A) Representative images showing YAP1 protein expression in human head and neck carcinoma tissues and tumor-adjacent normal tissues as analyzed by IHC. (a, b) Tumor-adjacent normal tissue. - (no stain) (c, d) Well-differentiated squamous cell carcinoma. + (low stain) (e, f) Well-differentiated squamous cell carcinoma. ++ (moderate stain) (g, h) Moderately differentiated squamous cell carcinoma. +++ (high stain) (B) The expression levels of key proteins in HNSCC cells were measured by Western blotting in the steady-state growth.

YAP1 protein expression was evaluated using IHC in head and neck carcinoma tissues and tumor-adjacent normal tissues. Representative results are shown in Fig. 1A. Our results show that YAP1 expression was significantly upregulated in carcinoma tissues compared to that in tumor-adjacent normal tissues (Table 2).

### Differential baseline expression of key genes in HNSCC cells

Fig. 1B shows the expression of key proteins in HNSCC cells in steady-state growth. YAP1 expression was non-significantly associated with HPV status, however, YAP1 expression in SCC-25 and UM-SCC-47 cell lines was higher than in the other HNSCC cell lines and YAP1 expression in 93-VU-147T cell line was lowest in the HNSCC cell lines tested. *CDH1* (E-cadherin gene) expression was higher in UPCI-SCC-090 and 93-VU-147T cells than in the other HNSCC cell lines. Snail expression was high in all HNSCC cell lines except UPCI-SCC-090 cell line, suggesting that the Snail may inhibit E-cadherin expression in HNSCC cells and that the expression levels of E-cadherin and Snail are inversely related. These results suggest that SCC-4, SCC-25, CAL-27, UM-SCC-47 and 93-VU-147T cells are epithelial-like cells with partial EMT, while UPCI-SCC-090 cells still maintain epithelial properties. Oct4 expression was high in all HNSCC cell lines except UPCI-SCC-090 and 93-VU-147T cell lines, suggesting that these cell lines lack stemness. HPV-negative HNSCC cells had relatively higher levels of EGFR expression than HPV-positive HNSCC cells, consistent with our previous results 15. PD-L1 expression was higher in UPCI-SCC-090 and 93-VU-147T cell lines than in the other HNSCC cell lines. The transcription levels of key genes in HNSCC cells (Figure S1) were consistent with the protein levels.

### The inhibitory effects of verteporfin on HNSCC cells were enhanced by light activation

**Figure 2:**
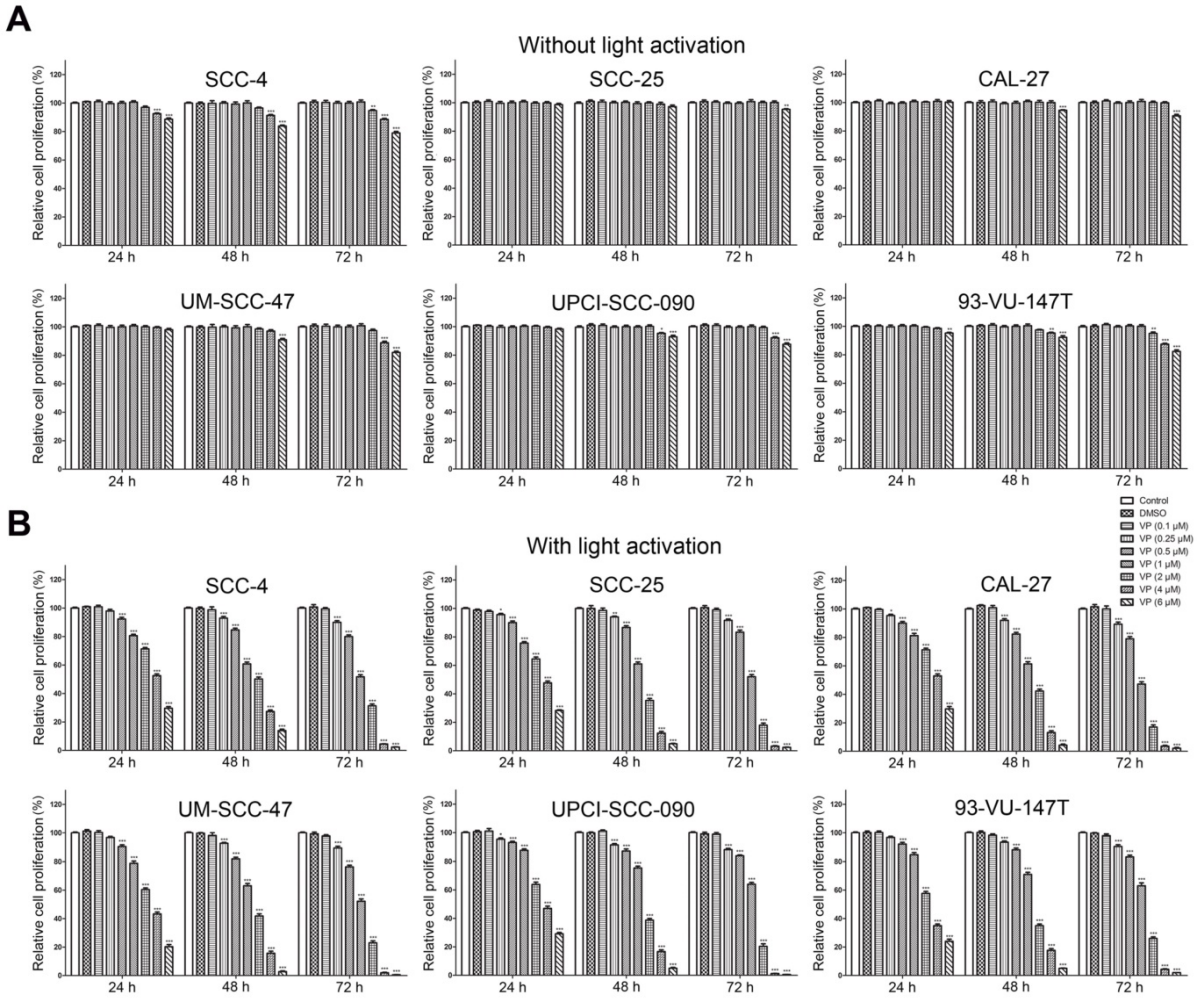
Inhibitory effect of VP on the proliferation of HNSCC cells. Inhibitory effects of VP on the proliferation of HNSCC cells without light activation (A) and with light activation (B) using the CCK-8 assay. The means±SEMs of three independent experiments are shown. *P < 0.05, **P < 0.01, ***P < 0.001 by Student's t-test

Fig. 2 shows the effects of VP on the proliferation of HNSCC cells with or without light activation by CCK-8 assay. VP exhibited minimal cytotoxicity to HNSCC cells at low concentrations (0.5 μM-4 μM) but some cytotoxicity at high concentrations (4 μM-6 μM) without light activation (Fig. 2A). However, VP significantly inhibited the proliferation of HNSCC cells in a dose-dependent manner with light activation (Fig. 2B). Our results showed that the response of HPV-positive HNSCC cells to VP was not appreciably different from that of HPV-negative HNSCC cells (Fig. 2B).

### Verteporfin induced apoptosis and G2-phase arrest in HNSCC cells

**Figure 3:**
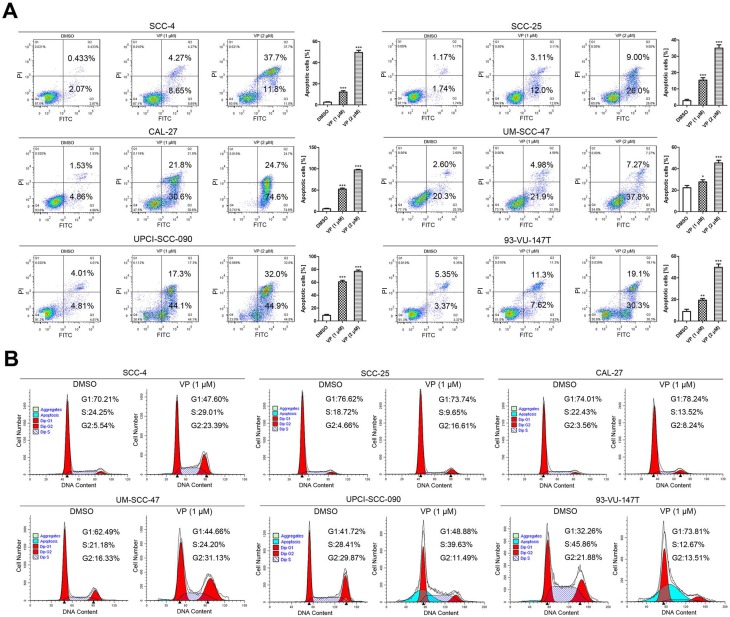
VP induces apoptosis and G2 arrest in HNSCC cells. (A) Cells were cultured for 24 h after VP light activation, and the apoptosis rates were determined by flow cytometry. The experiment was performed in triplicate, and the data are presented as the means±SEMs. *P < 0.05, **P < 0.01, ***P < 0.001 by Student's t-test (B) Flow cytometry showed that HNSCC cell growth was inhibited in the G2 phase following exposure to light activated VP for 24 h. The experiment was performed in triplicate, and the data are presented as the means±SEMs.

The apoptosis assays showed that the percentages of apoptosis in control SCC-4, SCC-25, CAL-27, UM-SCC-47, UPCI-SCC-090 and 93-VU-147T cells were nearly 2.5%, 2.9%, 6.4%, 22.9%, 8.8% and 8.7%; these percentages increased to 12.9%, 15.1%, 52.4%, 26.9%, 61.4% and 18.9% after treatment with 1 μM VP for 24 h and increased further to 49.5%, 35.0%, 99.3%, 45.0%, 76.9% and 49.4%, respectively, after treatment with 2 μM VP for 24 h. Histogram analysis indicates that the apoptotic cells are significantly increased in the VP treated groups relative to the control (Fig. 3A).

Fig. 3B shows that under normal conditions, the cell cycle distribution was different among these cell lines. Generally, the percentage of cells in G1 phase was higher in HPV-negative HNSCC cells than in HPV-positive HNSCC cells. When DNA is damaged, the cell cycle is arrested at the G1/S checkpoint or G2/M checkpoint for DNA repair; alternatively, cells will undergo apoptosis if repair efforts fail. Fig. 3B shows the cell cycle distribution of HNSCC cells after treatment with 1 μM VP for 12 h. G2 phase arrest was observed in most cell lines except UPCI-SCC-090 and 93-VU-147T. UPCI-SCC-090 and 93-VU-147T cells were arrested at the G1/S phase transition and in G1 phase, respectively. A noticeable sub-G1 population (apoptotic cells) was observed in UPCI-SCC-090 and 93-VU-147T cells.

### Verteporfin significantly inhibited colony formation by HNSCC cells

**Figure 4:**
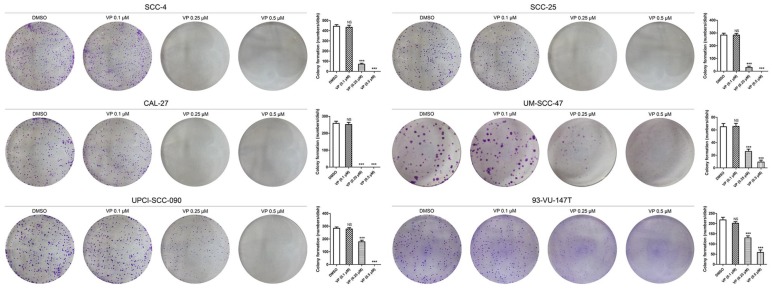
Inhibitory effects of VP on colony formation by HNSCC cells. After VP light activation, HNSCC cells were cultured for 10 days with the fresh medium. The data are presented as the means ± SEMs. ***P < 0.001 by Student's t-test

In the colony formation assay, equal numbers of viable HNSCC cells per well were seeded in 6-well culture plates before treatment with different concentrations of VP. The results showed that 0.25 μM and 0.5 μM VP significantly inhibited the colony forming capacity compared with DMSO treatment (Fig. 4).

### Verteporfin significantly inhibited the migration and invasion of HNSCC cells

**Figure 5:**
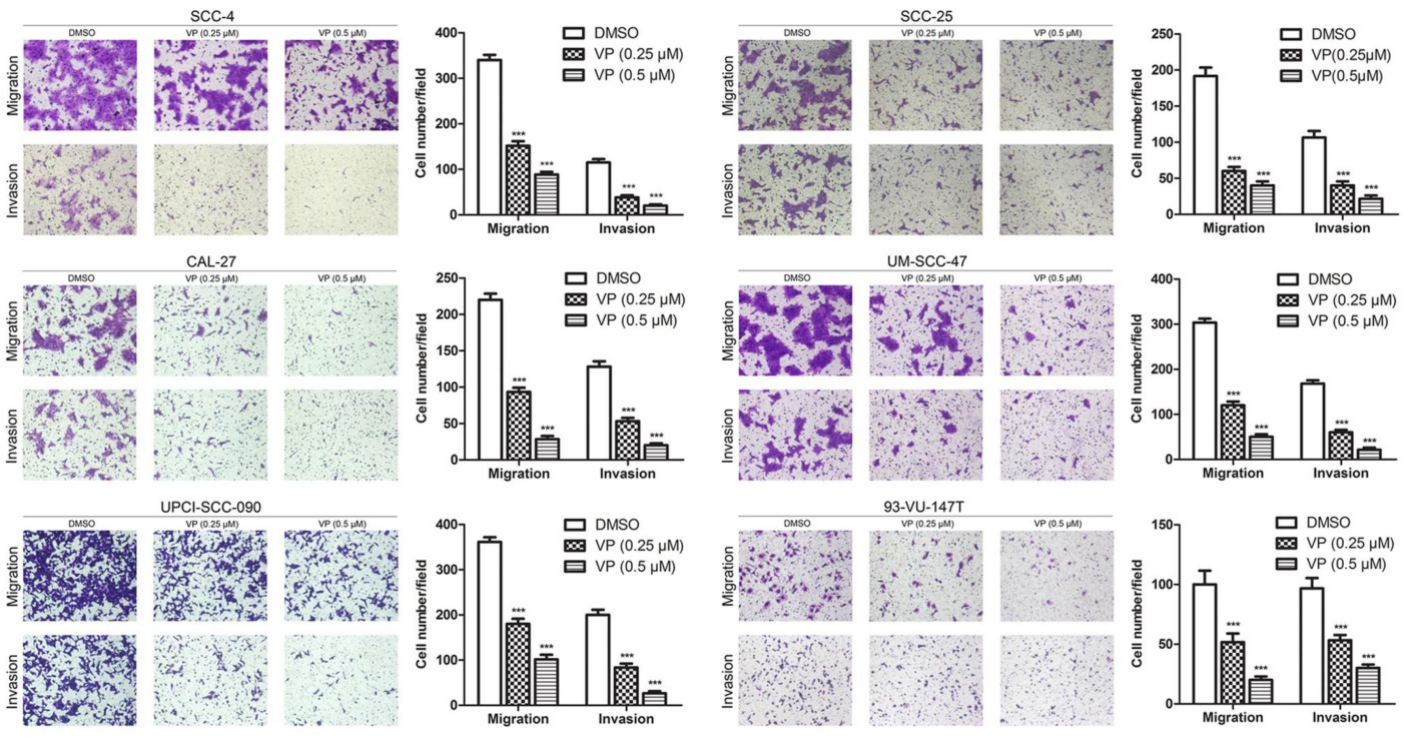
Inhibitory effects of VP on the migration and invasion of HNSCC cells. Cells were cultured for 24 h after VP light activation, and the extents of HNSCC cell migration and invasion were determined using a Transwell assay. The experiments were performed in triplicate, and the data are presented as the means±SEMs. ***P<0.001 by Student's t-test

The effect of VP on the migration of HNSCC cells was examined using the transwell migration assay. In the present study, the migration of cells treated with 0.25 and 0.5 µM VP was significantly decreased in a dose-dependent manner compared with the DMSO group cells (Fig. 5). Consistently, the results of the wound healing assay revealed that VP at concentrations of 0.25 µM and 0.5 µM significantly inhibited the migration of HNSCC cells compared with the DMSO group cells (Figure S2). Furthermore, the transwell invasion assays showed that cell invasion activity was significantly inhibited by VP in a dosage dependent manner (Fig. 5). These results clearly demonstrated that the HNSCC cell migration and invasion ability was inhibited by VP.

### Verteporfin altered the expression of key genes (proteins) in HNSCC cells

**Figure 6:**
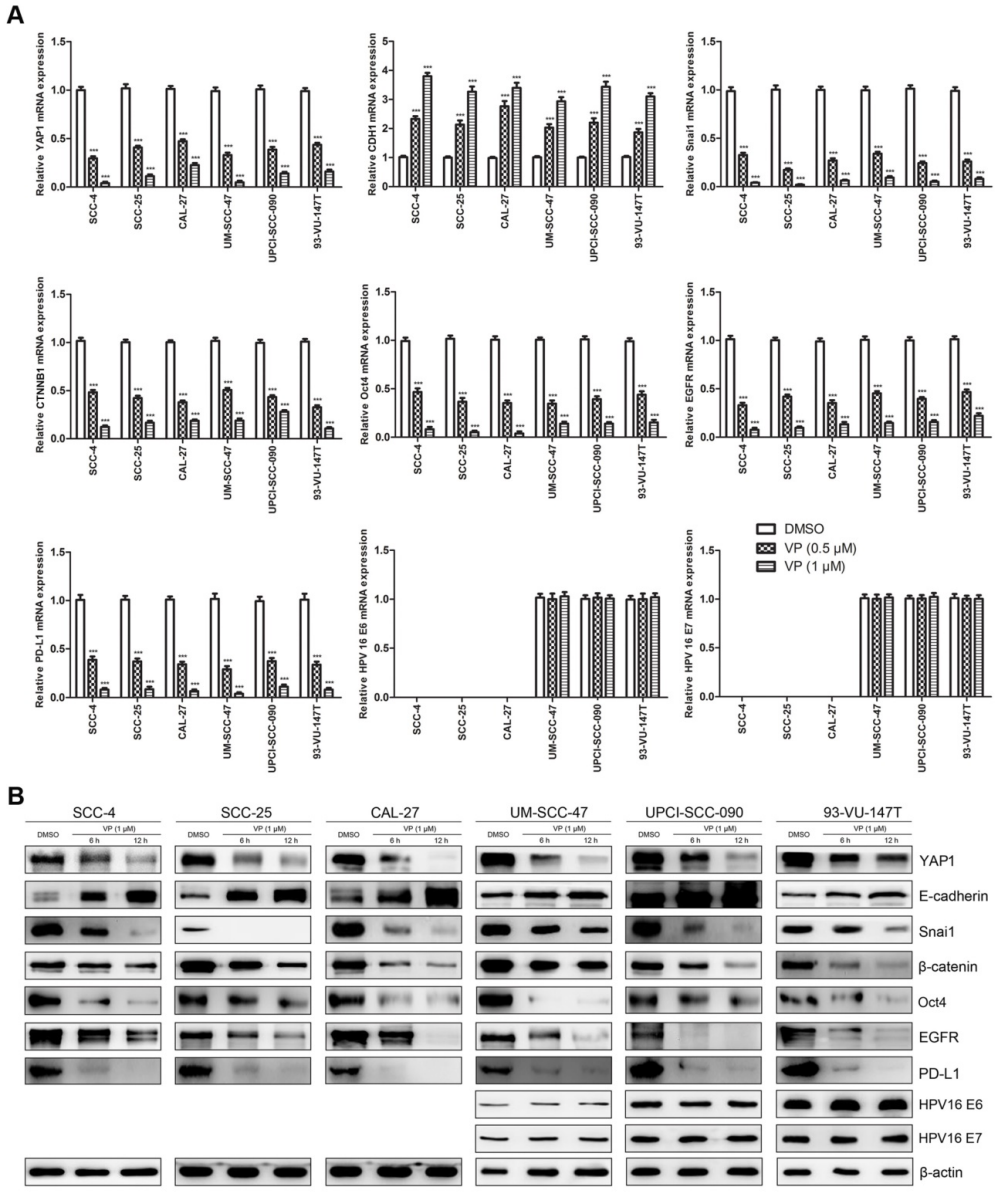
Effects of VP on mRNA and protein expression in HNSCC cells. (A) The transcription levels of key genes were assessed by RT-qPCR analysis of HNSCC cells treated with light activated VP for 12 h. The relative transcription levels of those genes were normalized to those of β-actin. The data are presented as the means ± SEMs. ***P < 0.001 by Student's t-test (B) The expression levels of key proteins were determined by Western blot analysis of HNSCC cells treated with light activated VP for 6 h and 12 h.

Fig. 6 shows the effects of VP on key genes in HNSCC cells at the mRNA (Fig. 6A) and protein (Fig. 6B) levels. VP treatment significantly decreased the expression of YAP1, Snai1, *CTNNB1* (β-catenin gene), Oct4, EGFR and PD-L1, whereas the expression of E-cadherin was upregulated in HNSCC cells. Our results showed that the response of HPV-positive HNSCC cells to VP was generally similar to that of HPV-negative HNSCC cells except E-cadherin. E-cadherin expression in SCC-4, SCC-25 and CAL-27 cells was increased by approximately 12-, 10- and 7-folds, respectively, whereas E-cadherin expression in UM-SCC-47, UPCI-SCC-090 and 93-VU-147T cells was only increased by approximately 2-, 2- and 3-folds, respectively, after treatment with 1 μM VP for 12 h compared with the control. However, the expression levels of HPV-16 E6 and E7 did not change with VP treatment.

### Verteporfin suppressed HNSCC cell xenograft growth

**Figure 7:**
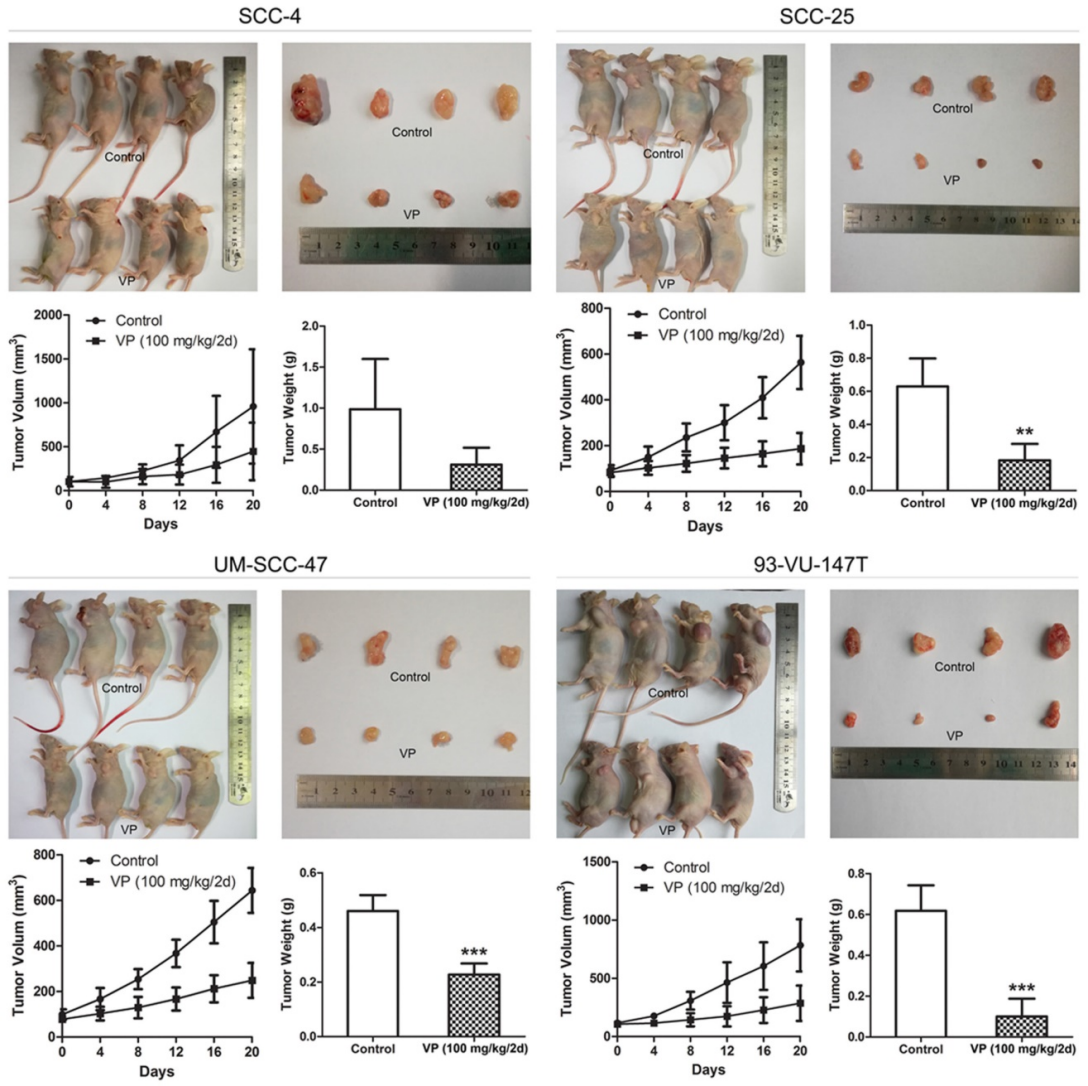
Inhibitory effects of VP on HNSCC cell xenograft growth. When the xenograft tumors attained a volume of ~80 mm^3^, the mice were treated with VP or vehicle every other day for 21 days by intraperitoneal injection. The tumor volumes and weights were determined after sacrifice. The data are presented as the means±SEMs. **P<0.01, ***P<0.001 by Student's t-test

After treatment with VP for 21 days, VP treatment significantly inhibited tumor growth compared to that of control tumors (Fig. 7) and tumor volume (mm^3^) in the SCC-4, SCC-25, UM-SCC-47 and 93-VU-147T cell models was declined from 959, 563, 644, 783 in the control groups to 447, 186, 249, 286 in the VP groups, respectively. This inhibitory effect was no obviously difference between HPV-positive xenografts and HPV-negative xenografts, consistent with that YAP1 expression was not closely associated with HPV status in HNSCC cells (Fig. 1B).

## Discussion

The cBioPortal online analysis tool (cBioPortal for Cancer Genomics) 16 shows that *YAP1* is frequently altered in different types of cancers according to data in The Cancer Genome Atlas (TCGA) database and head and neck carcinoma exhibits the second highest frequency of *YAP1* gene alteration 8. Consistent with previous reports 17, 18, our results showed that YAP1 expression was significantly upregulated in head and neck carcinoma tissues compared to that in tumor-adjacent normal tissues, suggesting that YAP1 overexpression is involved in HNSCC tumorigenesis.

The baseline expression levels of key genes differ among HNSCC cells (Fig. 1B), reflecting the different molecular characteristics of these cells. Our data showed that YAP1 expression was not appreciably different between HPV-positive and -negative HNSCC cells, consistent with previous reports 8, 19, suggesting that HPV oncoproteins may not influence YAP1 expression. Moreover, our data showed that YAP1 expression was highest in UM-SCC-47 cells, moderate in UPCI-SCC-090 cells and lowest in 93-VU-147T cells in the three HPV-positive HNSCC cell lines, consistent with a previous report, in which authors show that UM-SCC-47 cell line has *YAP1* gene amplification and 93-VU-147T cell line has *YAP1* gene deep deletion 19.

The anticancer effects of the photodynamic agent VP are reported to be different with light activation 12, 20, 21 and without light activation 22, 23. Our data showed that VP exhibited minimal cytotoxicity to HNSCC cells at low concentrations (0.5 μM-4 μM) without light activation, whereas the cytotoxic effects of VP on HNSCC cells were significantly higher at the same concentrations with light activation, indicating that light-induced activation is required for VP cytotoxicity. Reactive oxygen species (ROS) generated upon light-induced activation may account for this cytotoxic effect after the cellular uptake of VP 20, 24. Although we can't exclude the effect of ROS induced by VP after light activation on normal cells or tissues, in general normal cells may be capable of maintaining redox homeostasis under exogenous ROS 25 and tumor cells are more prone than normal cells to oxidative stress 26. Thus, our data suggest that light activation is necessary if VP is used in oncological therapy in the future.

Although differences in the cell cycle distribution between HPV-negative and -positive HNSCC cells were observed (Fig. 3B), the response of HPV-positive HNSCC cells to VP was not appreciably different from that of HPV-negative HNSCC cells. The only difference is that there was a sub-G1 peak in HPV-positive UPCI-SCC-090 and 93-VU-147T cells. The observed inhibitory effects of VP on HNSCCs are attributed to G2 or G1 phase arrest and apoptosis. Similar effects of VP have been reported, such as G1 phase arrest in pancreatic adenocarcinoma cells 13 and G2/S arrest in thyroid cancer cells 14.

The results of cell colony formation, migration and invasion assays showed that low concentrations of VP significantly inhibited the colony formation, migration and invasion of HNSCC cells, which indicates that VP inhibits EMT and stemness of HNSCC cells. Capacity of cell colony formation is generally considered to be associated with cancer stem-like cells, and the abilities of cell migration and invasion are considered to be linked to EMT. Previous studies have shown that VP can significantly inhibit cell adhesion and invasion of breast cancer cells 27 and reducing YAP protein suppresses migration and invasion of non-small cell lung cancer cells 28.

EMT and stemness are basic features of cancer stem cells, and YAP1 is involved in these processes 29. Our data showed that VP significantly attenuated the expression of genes related to EMT (*YAP1*, *Snail*, *CTNNB1* and *EGFR*) and stemness (*Oct4* and *YAP1*) and increased E-cadherin expression in HNSCC cells. The expression level of E-cadherin, a major interepithelial adhesion molecule, is closely related to EMT. Reduced or absent E-cadherin expression has been considered to be an initial step for the invasion and metastasis of many carcinoma cells 30. Snail has been demonstrated to repress the transcription of the *CDH1* gene. The loss of E-cadherin expression was mostly caused by Snail expression in our study, since the Twist and Snail are the most commonly expressed TFs in HNSCC 31, and the expression levels of Twist1 were unchanged in HNSCC cells after treatment with VP when E-cadherin was upregulated (Figure S3). Although the expression of E-cadherin was higher in UPCI-SCC-090 and 93-VU-147T cells than in the other HPV-negative HNSCC cells in steady-state growth (Fig. 1B), suggesting that HPV oncoproteins may influence E-cadherin expression, Fig. 6B showed that the upregulation of E-cadherin expression was more obvious in HPV-negative HNSCC cells than in HPV-positive HNSCC cells after VP treatment, suggesting that VP may be a novel drug inhibiting the invasion and metastasis of HPV-negative HNSCC.

The expression of a stemness marker Oct4 in 93-VU-147T cell line was lowest among all HNSCC cell lines, suggesting that this cell line lacks cancer stemness. Consistent with this finding, previous reports have shown that 93-VU-147T cells are the most sensitive one of tested HNSCC cell lines to therapies 15,32. Oct4 has been demonstrated to be a key regulator of stemness in HNSCC 33. YAP1 can regulate cancer cell EMT and stemness via the YAP1-Oct4-Sox2 signaling axis 34 by directly interacting with Oct4 through its WW domain 35. A previous study has reported that VP downregulates Oct4 expression in retinoblastoma cells 23.

β-Catenin plays two major roles in cells. In canonical Wnt/β-catenin signaling, it is the key effector responsible for transducing signals to the nucleus to trigger the expression of target genes, including genes orchestrating the EMT program. The second role of β-catenin is linked to E-cadherin in the formation of epithelial cell-cell adherens junctions. The triggering of EMT by β-catenin is well known 36. In fact, Hippo/YAP signaling exhibits multiple layers of interaction with Wnt/β-catenin signaling 37, 38, and both pathways cooperate to promote EMT and stemness. YAP has been reported to promote human glioma growth through partially enhancing Wnt/β-catenin signaling 38.

EGFR, a receptor tyrosine kinase, is of particular interest in HNSCC, as it is detectable in approximately 85% of HNSCCs 39. We found that the expression of EGFR seemed to be negatively associated with the expression of E-cadherin. The mechanism is somewhat complex and possibly operates through the activation of EGFR to decrease cell adhesion 40, or reduction of E-cadherin results in upregulation of *EGFR* transcription 41. EGFR signaling plays a significant role in EMT in HNSCC 42. Our data showed that VP inhibited EGFR expression, consistent with the results of Song et al. who showed that YAP1 induced *EGFR* transcription via a TEAD binding site in the *EGFR* promoter 43. They also found that VP great reduced xenograft growth of esophageal cancer JHESO cells, which express EGFR 43.

Our data showed that the expression of PD-L1, a ligand for the immune checkpoint protein programmed death 1 (PD1), was increased in UPCI-SCC-090 and 93-VU-147T cells, suggesting that HPV oncoproteins may influence PD-L1 expression in HPV-infected cells. The relationship between PD-L1 and HPV status in HNSCC is controversial. For example, Schoenfeld et al. reported that PD-L1 expression was associated with HPV status in oropharyngeal squamous cell carcinoma (OSCC) 44, whereas Kim et al. reported PD-L1 expression in the majority of OSCC patients regardless of HPV status 45. PD-L1 is physiologically expressed at low levels but can be highly expressed in neoplastic cells 46. *PD-L1* gene amplification and PD-L1 protein expression have been reported to be common events in squamous cell carcinoma of the oral cavity 47. Therefore, inhibited or reduced PD-L1 expression on cancer cells facilitates T cell activation and ameliorates the immunosuppressive microenvironment. The PD1 inhibitors pembrolizumab and nivolumab are currently approved for HNSCC therapy, and the PD-L1 inhibitors durvalumab, atezolizumab and avelumab are under evaluation in HNSCC 48. Some researchers recently reported that YAP regulates PD-L1 expression via binding to the *PD-L1* enhancer in lung cancer cells 49, 50. Our data showed that VP significantly downregulated the expression of PD-L1 in HNSCC cells, consistent with the results of Hsu et al. who reported that PD-L1 expression was correlated with YAP expression and VP downregulated PD-L1 expression in human malignant pleural mesothelioma 51.

Additionally, we examined the effect of VP on the expression of the *E6* and *E7* oncogenes in three HPV-positive HNSCC cell lines. However, VP did not change the expression of E6 and E7, suggesting that the anticancer effects of VP are mediated through an E6 and E7-independent mechanism in HPV-positive HNSCC cells.

In conclusion, our results demonstrated that VP inhibited the proliferation of HNSCC cells both *in vitro* and *in vivo*, and the inhibitory effects of VP on HNSCC cells were significantly enhanced by light activation *in vitro*. The anticancer effects of VP on HNSCC cells are mediated via the attenuation of the expression of genes related to EMT and stemness and the increase of E-cadherin expression. Furthermore, VP significantly inhibited the expression of immunosuppressive protein PD-L1 in HNSCC cells. These data indicate that VP is a potential therapeutic drug for HNSCC.

## Supplementary Material

Supplementary figures.

## Figures and Tables

**Table 1:** The primers of RT-qPCR

Gene	Forward (5ʹ-3ʹ)	Reverse (5ʹ-3ʹ)
YAP1	GCTACAGTGTCCCTCGAACC	TCCTTCCAGTGTTCCAAGGT
CTNNB1(β-catenin)	CATCTACACAGTTTGATGCTGCT	GCAGTTTTGTCAGTTCAGGGA
CDH1(E-cadherin)	CCGGGACAACGTTTATTACTAT	CATAGTCAAACACGAGCAGAGAAT
Snai1	CTCAAGATGCACATCCGAAGC	GCCTGGCACTGGTACTTCTTG
Oct-4	GTGCCGTGAAGCTGGAGAA	TGGTCGTTTGGCTGAATACCTT
EGFR	AGAGGATGTTCAATAACTGTGAGGTG	AGGGCAATGAGGACATAACCAG
PD-L1	GGTGCCGACTACAAGCGAAT	TAGCCCTCAGCCTGACATGTC
β-actin	CACCCAGCACAATGAAGATC	CTGATCCACATCTGCTGGAA

**Table 2:** YAP1 expression in 70 head and neck carcinoma tissues and 10 tumor-adjacent normal tissues.

	- Number (%)	+ Number (%)	++ Number (%)	+++ Number (%)	*P* value*
Tumor-adjacent tissues	5 (50)	5 (50)	0 (0)	0	
Carcinoma tissues	2 (2.9)	40 (57.1)	26 (37.1)	2 (2.9)	0.001

*P value is determined by Chi-square test.

## Abbreviations

DMSO: dimethyl sulfoxide
EGFR: epidermal growth factor receptor
EMT: epithelial-mesenchymal transition
HNSCC: head and neck squamous cell carcinoma
HPV: human papillomavirus
IHC: immunohistochemistry
Oct4: Octamer-binding protein 4
OSCC: oropharyngeal squamous cell carcinoma
PD1: programmed death 1
PD-L1: PD 1 ligand
ROS: reactive oxygen species
TEAD: TEA domain family transcription factors
TF: transcription factor
VP: verteporfin
YAP: Yes-associated protein
