# Supplementary material for: Verteporfin suppresses the proliferation, epithelial-mesenchymal transition and stemness of head and neck squamous carcinoma cells via inhibiting YAP1
Source: J Cancer. 2019 Jul 10;10(18):4196–207. doi: 10.7150/jca.34145 (PMC6691709; doi:10.7150/jca.34145)
Supplement: Supplementary file 1 — Supplementary figures. [file jcav10p4196s1.pdf]

Supplementary materials

Figure S1. Expression levels of key genes in HNSCC cells in the steady-state growth. The RT-qPCR results revealed the differential expression of *YAP1*, *CDH1*, *Snai1*, *CTNNB1*, *Oct4*, *EGFR*, and *PD-L1* in HNSCC cells. The relative mRNA levels of key genes were normalized to those of *β-actin*.

Figure S2. Inhibitory effect of VP on the migration of HNSCC cells. Cells were cultured for 24 h after VP light activation, and the HNSCC cell migration distance was determined using a wound-healing assay. The data are presented as the means ± SEMs. $^*P < 0.05$, $^{**}P < 0.01$, $^{***}P < 0.001$ by Student's t-test

Figure S3. Effects of VP on the expression of E-cadherin and Twist1 in HNSCC cells. The levels of E-cadherin and Twist1 proteins were determined by Western blot analysis of HNSCC cells treated with VP for 12 h.

Figure S1

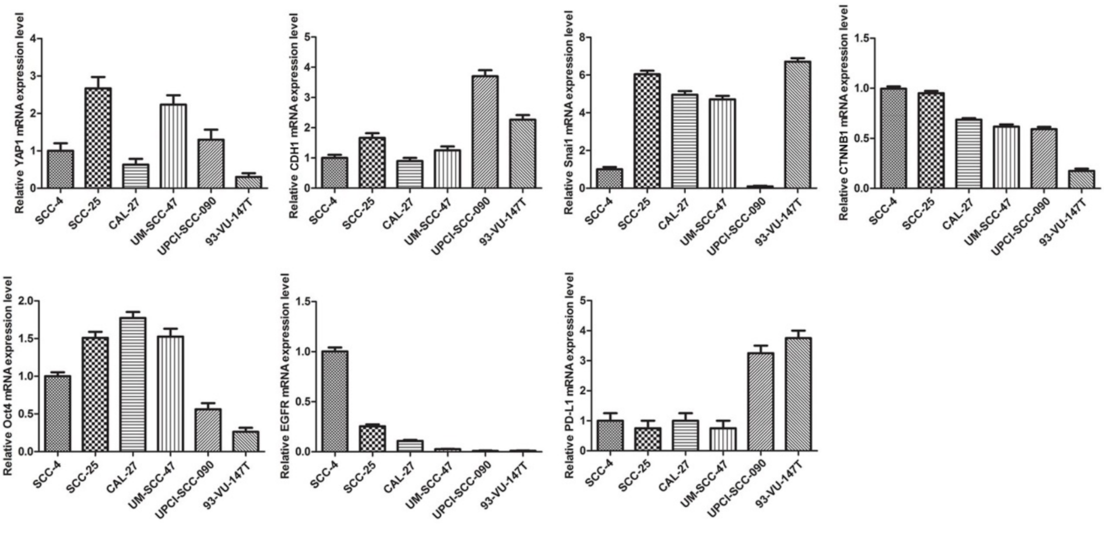
Relative YAP1 mRNA expression level
Relative CDH1 mRNA expression level
Relative Snai1 mRNA expression level
Relative CTNNB1 mRNA expression level
Relative Oct4 mRNA expression level
Relative EGFR mRNA expression level
Relative PD-L1 mRNA expression level
SCC-4
SCC-25
CAL-27
UM-SCC-47
UPCI-SCC-090
93-VU-147T

Figure S2

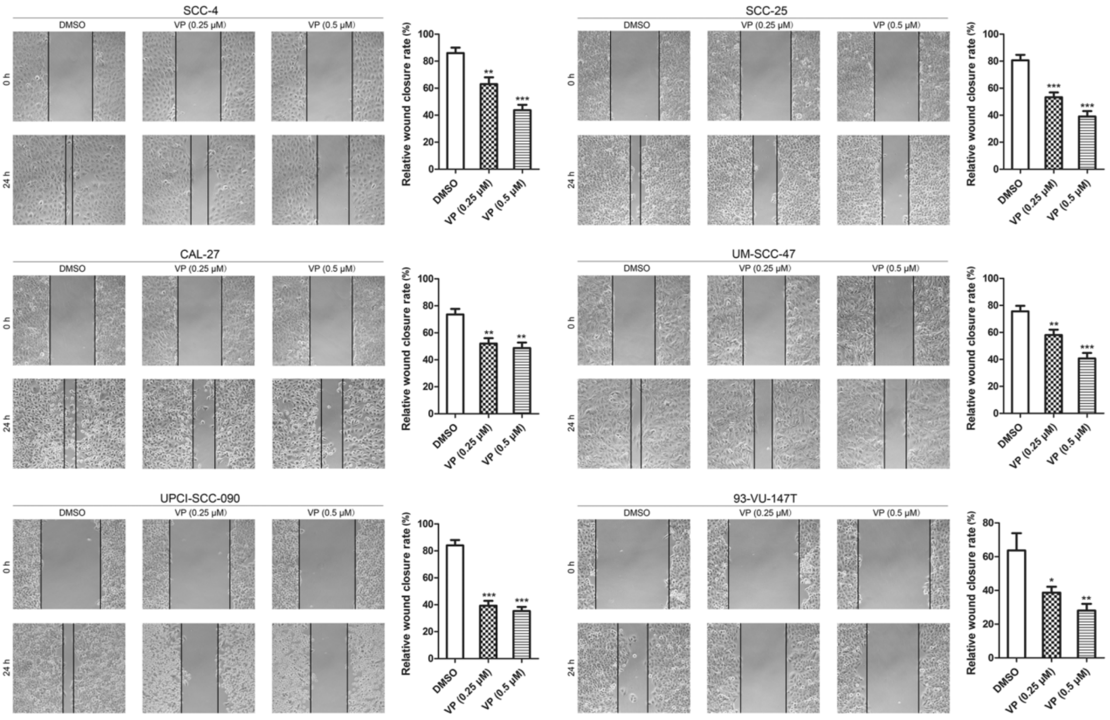
SCC-4
DMSO
VP (0.25 μM)
VP (0.5 μM)
0 h
24 h
Relative wound closure rate (%)
SCC-25
CAL-27
UM-SCC-47
UPCI-SCC-090
93-VU-147T

Figure S3

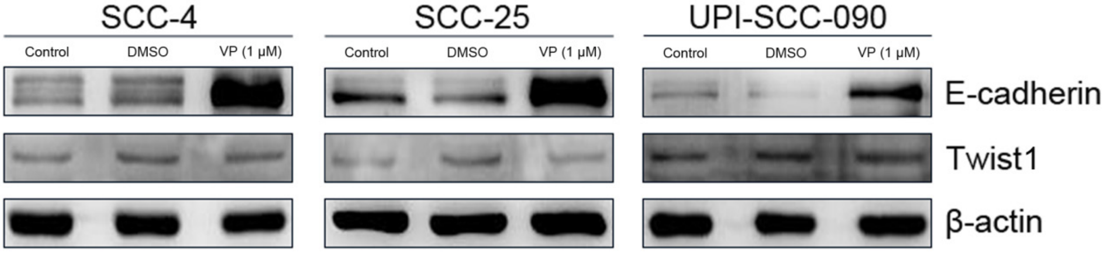
SCC-4
SCC-25
UPI-SCC-090
Control
DMSO
VP (1 μM)
Control
DMSO
VP (1 μM)
Control
DMSO
VP (1 μM)
E-cadherin
Twist1
β-actin
